# Severity of cystoid macular oedema in preterm infants observed using hand-held spectral domain optical coherence tomography improves weekly with postmenstrual age

**DOI:** 10.1038/s41433-023-02461-8

**Published:** 2023-03-16

**Authors:** Samira Anwar, Mintu Nath, Irene Gottlob, F. A. Proudlock

**Affiliations:** 1 Department of Ophthalmology, University Hospitals of Leicester NHS Trust, Leicester, UK; 2 University of Leicester Ulverscroft Eye Unit, Robert Kilpatrick Clinical Sciences Building Leicester Royal Infirmary, Leicester, UK; 3 Institute of Applied Health Sciences, Polwarth Building, University of Aberdeen, Aberdeen, Scotland

**Keywords:** Retinal diseases, Retina, Retina

## Abstract

**Objective:**

To investigate the relationship between cystoid macular oedema (CMO) measured in preterm infants using hand-held spectral domain optical coherence tomography (HH SD-OCT), with gestational age at birth (GA), birthweight (BW), diagnosis of retinopathy of prematurity (ROP) and the presence or absence of the external limiting membrane (ELM).

**Methods:**

We conducted a prospective mixed cross-sectional/longitudinal observational study of 112 participants (23 to 36 weeks GA; *n* = 25 with, and *n* = 87 without, CMO). Retinal images were acquired using 344 HH SD-OCT (*n* = 66 with and *n* = 278 without, CMO) between 31 to 44 weeks postmenstrual age (PMA). CMO type (‘fovea’ and ‘dome’) was measured using thickness, width, area and peak.

**Results:**

CMO was observed in 22.9% of preterm infants, and 19.2% of images. The mean values for  thickness, width, area and peak of ‘dome’ CMO were 128.47 µm (SD +/- 34.23), 3624.45 µm (SD +/- 1323.03), 0.49 mm^2^ (SD +/- 0.28) and 279.81 µm (SD +/- 13.57) respectively. The mean values for  thickness, width, area and peak of ‘fovea’ CMO were 64.37 µm (SD +/- 17.11), 2226.28 µm (SD +/- 1123.82), 0.16 mm^2^ (SD +/- 0.11) and 95.03 µm (SD +/- 26.99) respectively. Thickness, area width and peak were significantly greater for ‘dome CMO compared with ‘fovea’ CMO (*P* < 0.0001 for thickness, area and peak; *P* < 0.01 for width). Area and width significantly decreased with PMA for ‘dome’ and ‘fovea’ CMO (*p* = 0.0028; *p* < 0.001 respectively). No association was found between the presence of ROP and the detection of CMO or detection of CMO with absence of ELM.

**Conclusions:**

HH -OCT in preterm infants demonstrates that the severity of CMO appearance improves each week for both fovea and dome CMO.

## Introduction

Ocular imaging using spectral domain optical coherence tomography (SD-OCT) in infants has revealed retinal details that are invisible with conventional examination [1–4]. A feature observed in many preterm infants is the presence of intraretinal cystic changes centred at the fovea described as cystic macular oedema (CMO) and visualised on SD-OCT as hyporeflective spaces within the retina [5–13]. To date, two patterns of CMO have been described, characterised by the presence or absence of the foveal pit: loss of the foveal depression due to distortion of the foveal contour from large intraretinal cystic spaces (‘dome CMO’) or preservation of the foveal pit with increased inner nuclear layer (INL) thickening from multiple cystic spaces (‘fovea CMO’) [6, 8, 9]. Dome CMO has not been reported in term-born infants [13] suggesting that dome CMO may be more likely to occur in immature retina.

The exact mechanism of infant CMO is currently unknown. Studies report that between 15% to 72% of preterm infants demonstrate CMO on at least one SD-OCT imaging session from 32 to 43 weeks postmenstrual age (PMA) [6–8]. There are fewer reports of CMO in term-born infants [13, 14]. It is thought that CMO does not resolve before 36 weeks PMA if CMO is found between 30 and 35 weeks PMA [6] and has disappeared at 52 weeks PMA [8].

The severity of CMO as measured by central foveal thickness (CFT) may be associated with an increased stage of retinopathy of prematurity (ROP) [8–10]. Equally there are reports of no such association [15] and CMO may be detected before and after treatment for ROP [6, 7, 12, 16].

CMO has not been associated with birth weight (BW), sex, ethnicity [7, 12, 16, 17] or systemic preterm neonatal risk factors such as sepsis, necrotising enterocolitis, periventricular leukomalacia, hydrocephalus or immature lung disease [6, 12]. However, a report by Wong et al. does suggest an association with intraventricular haemorrhage [15].

The significance of CMO on vision and development in infants is poorly understood making it an important area for investigation. In preterm infants with CMO, the development of the photoreceptor layer at the foveal centre by 42 weeks PMA is delayed using SD-OCT imaging [13] while at 3 months corrected age, reduced visual acuity and greater hyperopia is demonstrated for preterm infants with CMO compared with preterm infants with no CMO detected [18]. Preterm infants with CMO may also have worse neuro-developmental speech and language outcomes in early childhood between 18 of 24 months of corrected age [17].

Adult macular oedema is primarily pathological and several hypotheses regarding the pathophysiology of CMO are based on aetiology such as a breakdown of the blood retinal barrier (BRB) [19] or vascular retinopathies e.g. diabetes, branch retinal vein occlusion [20, 21].

Although the pathogenesis of CMO may be different in preterm infants, we hypothesised that disruption of the blood retinal barrier (BRB) due to absence of the external limiting membrane (ELM) may be associated with an increased likelihood of CMO detected in preterm infants. Additionally, we also investigated the severity of CMO in preterm infants seen  using hand held SD-OCT imaging (HH SD-OCT) with gestational age (GA), birth weight (BW), PMA, and diagnosis of ROP.

## Methods

The study was conducted in accordance with the tenets of the Declaration of Helsinki and approved by a Local Ethics Committee (NRES committee, Nottingham, East Midlands, United Kingdom). Patients were recruited from the Leicester Royal Infirmary neonatal and maternity unit, United Kingdom. All preterm babies from 31 to 44 weeks PMA who required ROP screening were eligible for inclusion in the study. Any abnormal ocular examinations, (other than a diagnosis of ROP stages 1-3), treated ROP, and ROP stages 4 or 5, were exclusion criteria. For screening and imaging, ROP was defined as stages 1–3 using the UK guidelines [22] and eyes were dilated using Cyclomydril® eye drops. Optical coherence tomography (OCT) scanning was conducted in both eyes using a portable spectral domain noncontact high-resolution hand-held OCT (HH SD-OCT) (Envisu C-Class, Leica Microsystems, Wetzlar, Germany).

Imaging was performed at 1 to 2 weekly intervals in eligible preterm infants from April 2012 until July 2014. The raw data consisted of a video centred at the foveal location and a minimum of five videos were taken of each infant for each eye at each imaging session. Scans were optimised for obtaining a single high-quality scan at the central retina and the lateral distance settings were corrected to account for the smaller axial lengths in the infant population using a conversion table according to PMA and GA from the data presented by Maldonado et al. [23].

At analysis, successful identification of the foveal centre was achieved by examining five uninterrupted B scans on either side of the B scan with the deepest point in the central retina [24]. Images with intraretinal hypo-reflected spaces distorting the foveal location and highly reflective intervening septae along with vertical expansion of the foveal location either with or without loss of the foveal contour were defined as CMO. These were then further divided into two groups depending on the presence or absence of the foveal depression (‘fovea’ CMO and ‘dome’ CMO respectively) [3, 6, 8, 11].

**Fig. 1:**
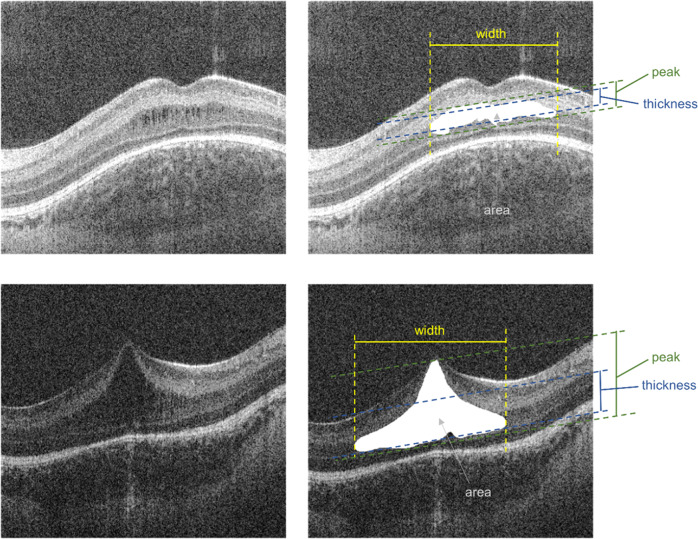
Hand-held spectral domain optical coherence tomography image of a preterm infant. Example of foveal hyporeflective space for analysis with measurements. Fovea CMO is shown in the top panel, dome CMO is shown below.

An example of CMO from the study group is shown in Fig. 1 which demonstrates hyporeflective spaces in the retina of two preterm infants.

For the purposes of the study, one single image for each infant at each imaging session defined as CMO was included in the analysis. Right and left eyes were included. Duplicate images were discarded. For each infant demonstrating CMO and weekly repeated images, the foveal location was matched as closely as possible when manual segmentation was undertaken to delineate the affected spaces involving the fovea and surrounding retina consistently.

The outline of the hyporeflective space was manually segmented using ImageJ (United States National Institutes of Health, Bethesda, MD, https://imagej.nih.gov/ij/, downloaded on December 2013) after inspection of the image and shown in white (Fig. 1). Data were saved as text files and a customised macro using Excel (Microsoft Windows®) was used to sum up the number of white pixels in each A-scan to calculate thickness, width, area and peak after incorporating an axial length correction for PMA based on previous literature [23].

The parameters are indicated in Fig. 1 (right column) where the width is the extent of the CMO along the x axis, thickness is the mean extent of the CMO along the y axis, i.e. in each A-scan containing the CMO, area is the sum of all the white pixels segmented and peak is the most anterior point of the CMO relative to the most posterior point after adjusting for the line through the posterior profile of the CMO.

Image data were recorded according to the presence or absence of the ELM, the type of CMO (dome, or fovea), diagnosis of ROP at the time of image acquisition, in addition to demographic data (sex, GA, BW, right or left eye, ethnicity, multiple births).

### Statistical analysis

To assess different risk factors associated with the incidence of CMO, we modelled the presence of CMO using a generalised linear mixed model with a logit link function. To account for the multiple measurements on the same subject, we incorporated the random intercept of the individual infant. Due to the high correlation between gestational age (GA) and birth weight (BW), we fitted two different models incorporating either GA or BW.

Both models included relevant individual-level predictors; like PMA, ELM (presence and absence), eye (right and left), ROP status (presence and absence at the time of image acquisition), sex (male and female), multiple births (yes and no) and ethnic group (Caucasian, Non-Caucasian) in our infants with and without ROP where we found a high correlation coefficient between GA and BW (*r* = 0.70, standard error = 0.08), we fitted separate models for all response variables incorporating either GA or BW as a predictor to adjust for the effects of GA or BW with either GA or BW in the model.

To evaluate the CMO appearance (fovea or dome) for different retinal characteristics (i) thickness, (ii) width, (iii) area (square root transformation), and (iv) peak of the CMO, we fitted separate linear mixed models for each of the retinal characteristics with the individual infant as a random effect. The model also included other predictor variables as indicated above, and separate models incorporating either GA or BW.

The linear mixed model was fitted using the R package lme4 (version 1.1-27). All statistical analyses were conducted in R statistical computing environment (version 4.0.3).

## Results

### Differences between preterm infants with CMO and without CMO

The differences in mean GA, BW and PMA are shown in Table 1 for participants with (*n* = 25, 22.3%) and without CMO (*n* = 87, 87.7%) of infants and in Table 2 for images, based on presence/absence of ROP, sex, multiple births, ethnicity (Caucasian or not) and presence/absence of ELM. The total number of images acquired that were suitable for analysis was 344 of which 66 (19.2%) were observed with CMO.

**Table 1:** Participant characteristics.

		CMO no	CMO yes
*Number of infants*			
Total 112		87	25 (22.3%)
Male	61	47	14 (22.9%)
Female	51	40	11 (21.6%)
Caucasian	60	46	14 (23.3%)
Non-Caucasian	52	41	11 (21.1%)
Single birth	90	75	15 (16.6%)
Multiple birth	22	12	10 (45.4%)
Mean (±SD) GA, BW, PMA			
GA (weeks)		28.2 (2.6)	29.8 (2.6)
BW (grams)		1085.7 (427.7)	1308.1(443.1)
PMA (weeks)		36.2 (2.6)	36.8 (2.4)

*GA* gestational age, *BW* birthweight, *PMA* postmenstrual age, *ELM* external limiting membrane, *CMO* cystoid macular oedema.

**Table 2:** Image characteristics.

		CMO no	CMO yes
*Number of images*			
Total 344		278	66 (19.2%)
Right eyes	176	132	44 (25%)
Left eyes	168	146	22 (13%)
NonROP	206	173	33 (16%)
ROP	138	105	33 (23.9%)
CMO fovea		_	32
CMO dome		_	34
ELM absent	244	195	49 (20.1%)
ELM present	100	83	17 (17%)

*GA* gestational age, *BW* birthweight, *PMA* postmenstrual age, *ELM* external limiting membrane, *CMO* cystoid macular oedema.

We found no associations related to GA (or BW), PMA, eye, ROP status, sex, ELM, ethnicity, or multiple birth to either the presence or absence of CMO. The results of the model including GA as a predictor are shown in Supplementary Table 1.

The mean (±standard deviation), respectively, for CMO and non-CMO participants was: 29.8 weeks (±2.6) and 28.2 weeks (±2.6), for GA and 1308.1 (±443.1) and 1085.7 g (±427.7) for BW.

In Table 2, CMO images, the breakdown was as follows; ROP: *n* = 22, (17.3% of all ROP images); non-ROP: *n* = 44 (20.3% of all non-ROP images), CMO with ELM present: *n* = 17 (17.0% of all ELM present images), and CMO with ELM absent: *n* = 49 (20.1% of all ELM absent images).

With non-CMO the breakdown was as follows; ROP: *n* = 105, (82.7% of all non-ROP images), non-ROP: *n* = 173, (79.7% of all ROP images); non-CMO with ELM present: *n* = 83 (83.0% of all ELM present images), and non-CMO with ELM absent: *n* = 195 (79.9% of all ELM absent images).

### Differences between CMO sub-type (fovea and dome)

Sixty-six images were acquired from 25 infants with CMO. Thirteen infants showed only fovea CMO, 2 had fovea initially which later developed dome CMO in both eyes, a further 2 had fovea CMO initially which later developed dome CMO in only one eye (right) i.e. both dome and fovea CMO was seen at the same imaging session and the remaining 8 infants showed only dome CMO. The number of images with a ‘fovea’ appearance of CMO images was 32, whereas the number of images with a ‘dome’ dome appearance was 34. The number of imaging sessions per infant is presented in Supplementary Table 2. For each preterm infant with CMO, only one image for right and/or left eye was selected for analysis for a particular session.

In Table 3 the differences in mean GA, BW and PMA are shown for infants with fovea and dome CMO along with the numbers of participants and images acquired based on the individual-level predictors for four retinal parameters. We found no associations between CMO appearance (fovea or dome) and eye, ROP status, sex, multiple births, ethnicity or presence/absence of the ELM.

**Table 3:** Mean differences for infants with fovea and dome Cystoid Macular Oedema along with the numbers of participants and images.

Cystoid macular oedema	Dome		Mixed	Fovea	
	Mean	SD		Mean	SD
Thickness (µm)	128.47	34.23		64.37	17.11
Width (µm)	3624.45	1323.03		2226.28	1123.82
Area (mm^2^)	0.49	0.28		0.16	0.11
Peak (µm)	279.81	13.57		95.03	26.99
Gestaional Age (weeks)	30.62	1.49		29.36	3.12
Birth Weight (g)	1512.78	460.11		1206.56	418.37
Postmenstrual Age (weeks)	38.03	2.84		37.72	2.39
Eye	Left	Right		Left	Right
No of images	15	19		18	14
No of participants	8		4	13	
ROP	Present	Absent		Present	Absent
No of images	8	26		14	18
No of participants	4	9		7	11
Sex	Female	Male		Female	Male
No of images	20	14		14	18
No of participants	8	5		6	11
Multiple births	Yes	No		Yes	No
No of images	12	22		13	19
No of participants	5	8		7	10
Caucasian	Yes	No		Yes	No
No of images	27	7		17	15
No of participants	9	4		8	9
ELM	Present	Absent		Present	Absent
No of images	4	30		13	19
No of participants	4	12		10	11

*SD* standard deviation, *ELM* external limiting membrane, *ROP* retinopathy of prematurity.

The only predictors associated with these retinal characteristics were PMA and CMO appearance, and reduced statistical models including only these two predictors are presented in Table 4. Supplementary Fig. 1 represents the mean changes (±95% Confidence Intervals) in CMO thickness, width, area and peak with PMA for fovea and dome appearance of CMO based on the models.

**Table 4:** Results of changes with Postmenstrual age (PMA) for fovea and dome cystoid macular oedema (CMO) characteristics.

	Estimate	Standard error	Degrees of freedom	*t* value	*P* value
**A. Thickness**					
PMA (weeks)	−2.39	1.32	62.89	−1.82	0.073
Appearance of CMO (fovea/dome)	−49.85	6.92	62.48	−7.21	<0.0001
**B. Width**					
PMA (weeks)	−161.75	51.83	59.50	−3.12	0.0028
Appearance of CMO (fovea/dome)	−752.98	271.26	58.04	−2.78	0.0074
**C. Area (square root)**					
PMA (weeks)	−26.01	6.74	55.16	−3.86	<0.001
Appearance of CMO (fovea/dome)	−185.43	35.14	53.40	−5.28	<0.0001
**D. Peak**					
PMA (weeks)	−4.69	3.69	61.20	−1.27	0.208
Appearance of CMO (fovea/dome)	−135.85	19.36	60.04	−7.02	<0.0001

All four measures were significantly larger for dome appearance compared to fovea appearance of CMO (*P* < 0.0001 for thickness, area and peak; *P* < 0.01 for width). All four measures showed a reduction with increasing PMA, although this was only statistically significant for width and area (*P* < 0.01 and *P* < 0.001, respectively).

## Discussion

We attempted to objectively measure preterm infant CMO to quantitively explore possible risk factors associated with two types of CMO (fovea and dome) previously described in the literature using hand held spectral domain OCT. In our study, preterm infants with CMO were on average born later and heavier at birth than those without CMO, but these differences were not statistically significant. In preterm infants demonstrating CMO, the only predictor of change was PMA, when CMO width and area decreased with each week. This suggests that increasing maturity of the central retina is one factor in the improvement of the foveal appearance following detection of CMO in infants. Few studies have reported the dynamic changes of preterm infant CMO with time.

Vinekar et al. observed that 10 preterm infants with CMO at ROP screening demonstrated normal foveal shape at 52 weeks PMA [18]. Our study suggests that this change occurs much earlier.

In our study population, preterm infants with CMO were older and heavier at birth than those with no CMO in our study. Although statistical modelling found no significant association between GA or BW with CMO, similar findings have been reported previously [8, 17]. However, this is in contrast with other authors who suggest that younger born infants are more likely to have CMO [6, 7, 9, 12].

Adult retinal histology shows cystic spaces are found in several retinal layers including ganglion cell, outer & inner nuclear, inner plexiform (GCL, ONL, INL, IPL Henlè’s fibres) [25, 26]. The exact underlying pathogenesis is unknown but a multifactorial aetiology is suspected [27, 28] with disruption of the blood retinal barrier (BRB), and damage to Müller cells considered to be likely associations [19, 28, 29]. Accumulation of protein results in oncotic pressure attracting water and the greatest oedema will be adjacent to where either fluid leaks for example, near capillaries. In adults, swelling of Müller cells has been shown using electron and light microscopy in cases of macular cystoid oedema [29, 30]. CMO appears petaloid on fundus fluorescein angiography (FFA) due to the vertical structure of the Müller cells that become stretched as a result. However, CMO on OCT without FFA leakage is also reported in adults who undergo taxane chemotherapy [31] and Vitamin B3 (Niacin) in the treatment of high cholesterol [32]. The underlying hypothesis is that these agents are toxic to Müller cells without disrupting the blood retinal barrier (BRB) [33].

Bilateral infant cystoid maculopathy has been described at postmortem reporting the location of the cystic spaces as the inner, outer and retinal nerve fibre layers [34]. In this report, Müller cells were stretched across the cavitation with the cells vertically lining the cavities in a ‘pillar’ like fashion.

Using SD-OCT, Bondalapati et al. [12] reported 4 preterm infants with cystic spaces involving the GCL. However, CMO in preterm infants has mostly been observed in the INL [6]. The INL has several cell types including Müller cells which span the entire width of the neurosensory retina, forming the internal and external (inner and outer, respectively) limiting membranes. It is possible that infant CMO may result from swelling of Müller cells themselves [29, 35]. Stretching of the Muller cells would result in the characteristic features found on OCT imaging as illustrated in Fig. 1.

The ELM which also forms part of the BRB provides structure to the retina [36]. Our study hypothesis was that the CMO in preterm infants could relate to the absence i.e. immaturity of the ELM at the fovea. However, we did not find a significant difference with either presence of absence of the ELM, between either the infants with and without CMO, or for infants with CMO, between the type of CMO. A report of FFA in a single preterm infant with macular oedema imaged at 49 weeks PMA, did not detect fluorescein leakage [37]. Infant CMO may therefore be similar to adult taxane maculopathy where Müller cell swelling can occur without BRB disruption and by extension unrelated to ELM absence or presence.

Higher stage of ROP has been correlated with CMO [8, 9] and could result from the increased levels of VEGF associated with Müller cell physiology [38]. We could not assess the risk with the stage of ROP due to the small numbers of CMO infants with ROP.

## Limitations

The small number of infants with CMO, especially dome CMO with the absence and presence of ELM, limits the evaluation of ELM association with CMO status. Also, we had only four infants who changed from fovea to dome (not vice versa) which limited the scope to assess if fovea CMO was more likely to develop into dome CMO or the other way around. Increased participant numbers would allow for predictive modelling regarding the differences between CMO type with respect to variables such as the ELM, ethnicity, multiplicity of birth and PMA. Further limitations include the lack of data on maternal or infant systemic medical history which might be relevant in the development of CMO [15, 17].

## Conclusion

Objective measurements of preterm infant CMO (width and area) show dynamic changes with time suggesting increased retinal maturity is a factor in the improved appearance of the fovea following CMO detection. Although we did not find a significant association with GA, this observation is in keeping with the fewer reports of CMO presence in term-born infants compared with preterm infants. The reported risk factors for the development of CMO in preterm infants, including ROP, remain inconclusive in our investigation.

## Summary

### What was known before

- Cystoid macular oedema is present in some preterm infants. Cystoid macular oedema in preterm infants may be associated with neuro-developmental outcome. The mechanism of cystoid macular oedema in preterm infants is unknown.

### What this study adds

- Cystoid macular oedema in preterm infants improves with postmenstrual age following birth suggesting retinal maturity is a factor and it reduces earlier than has previously been reported.

### Supplementary information

Supplementary Table 2

Supplementary Figure 1

Supplementary Table 1

## Data Availability

The data that support the findings of this study are not openly available due to sensitive human data and are available upon reasonable request to the Ulverscroft Eye Unit, where the data is stored in a controlled access repository.
