# Supplementary material for: Severity of cystoid macular oedema in preterm infants observed using hand-held spectral domain optical coherence tomography improves weekly with postmenstrual age
Source: Eye (Lond). 2023 Mar 16;37(14):3009–14. doi: 10.1038/s41433-023-02461-8 (PMC10516860; doi:10.1038/s41433-023-02461-8)
Supplement: Supplementary file 2 — Supplementary Figure 1 [file 41433_2023_2461_MOESM2_ESM.docx]

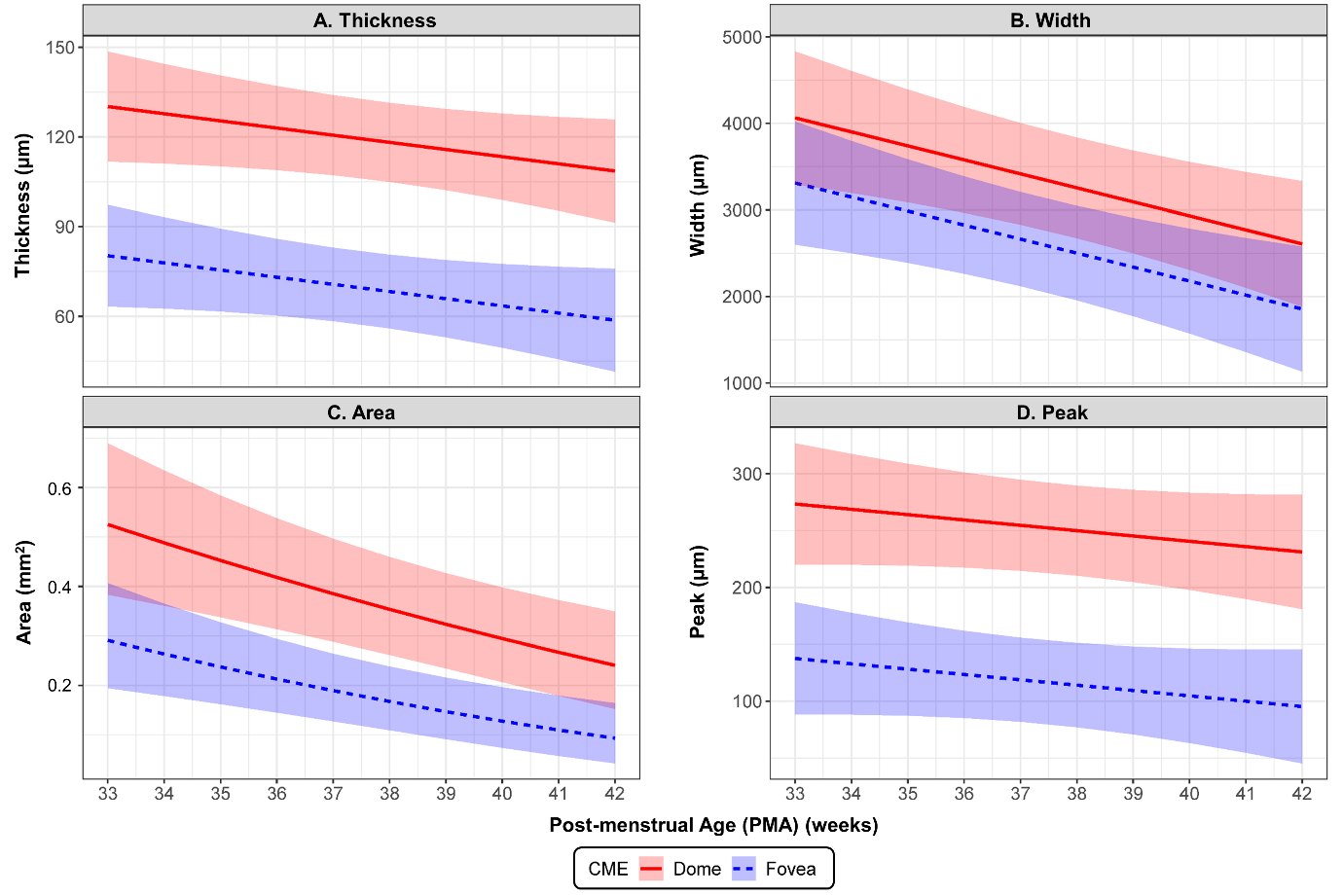


**Supplementary Figure 1** The mean changes (± 95% Confidence Intervals) in CME thickness, width, area and peak with PMA for fovea and dome appearance of CME.
